# MiR-503逆转肺癌耐药细胞株A549/DDP的耐药性及其机制研究

**DOI:** 10.3779/j.issn.1009-3419.2014.01.01

**Published:** 2014-01-20

**Authors:** 毅 武, 丽丽 郭, 京豪 刘, 仁旺 刘, 明辉 刘, 军 陈

**Affiliations:** 300052 天津，天津医科大学总医院肺部肿瘤外科，天津市肺癌研究所，天津市肺癌转移与肿瘤微环境实验室 Department of Lung Cancer Surgery, Tianjin Lung Cancer Institute, Tianjin Key Laboratory of Lung Cancer Metastasis and Tumor Microenvironment, Tianjin Medical University General Hospital, Tianjin 300052, China

**Keywords:** 微小RNA, 肺肿瘤, 耐药, MicroRNA, Lung neoplasms, Drug-resistant

## Abstract

**背景与目的:**

临床上肺癌细胞往往出现对顺铂的耐药性，因此探讨肿瘤细胞的耐药机制，开发新的逆转耐药性的方法，对提高临床患者的受益有十分重要的意义。miRNA可通过其调控的目标基因，对多种与肿瘤细胞失控生长、抗凋亡、迁移和侵袭，甚至是肿瘤细胞对药物治疗的应答产生调控作用。本实验旨在探讨miR-503对肺癌顺铂耐药细胞株A549/DDP的耐药性逆转及其相关作用机制。

**方法:**

应用MTS法检测miR-503对A549/DDP细胞顺铂耐受性的影响，流式细胞术检测肿瘤细胞凋亡率以及胞内罗丹明-123（Rhodamine-123, Rh-123）含量的变化，Western blot法和Real time PCR检测肿瘤细胞多药耐药蛋白MDR1、MRP1、Survivin和Bcl-2蛋白表达，以及Akt磷酸化的变化，应用双萤光报告基因技术检测细胞NF-κB和AP-1转录活性。

**结果:**

与对照细胞组相比较，miR-503转染A549/DDP细胞株后，可明显增加细胞对顺铂的敏感性，使耐药逆转倍数增加为2.48倍，Rh-123含量升高2.49倍，细胞凋亡率提高10.3倍；在转录水平检测发现，与对照组相比较，miR-503转染的细胞中MDR1、MRP1、ERCC1、Survivin及Bcl-2等与肿瘤耐药相关基因的mRNA表达水平明显下调，而RhoE mRNA表达水平则明显升高（*P* < 0.05）；进一步在蛋白水平亦证实MDR1、MRP1、ERCC1、Survivin、Bcl-2以及p-Akt的表达明显下降，RhoE的表达明显上升。

**结论:**

miR-503可逆转A549/DDP对顺铂的耐药性，这一作用可能与抑制药物外排，负调控肿瘤耐药相关蛋白的表达，促进细胞凋亡有关。

## Abstract (en)

**Background and objective:**

Cisplatin-resistance in lung cancer cells is general in clinic, hence it is significant to investigate the mechanisms of cisplatin-resistant and develop new methods of reversing drug-resistance. Recent researches showed that miRNA could regulate cell growth, apoptosis, migration and invasion even in drug therapy in cancer by its target gene. The aim of this study is to investigate the effects and molecular mechanisms of miR-503 on reversing the cisplatin-resistance in lung cancer DDP-resistant cell line A549/DDP.

**Methods:**

MTS assay was employed to determine the effect of miR-503 on A549/DDP' sensitivity to cisplatin. Apoptosis rate and intracellular concentration of rhodamine-123 (Rh-123) were determined by flow cytometry, the expression of multi-drugs resistant proteins MDR1 and MRP1, ERCC1, RhoE, Survivin and Bcl-2 were determined by Western blot and real time PCR. The phosphorylation of Akt was analyzed by Western blot, the transcriptional activities of NF-κB and AP-1 were detected by dual-luciferase reporter gene systems.

**Results:**

MiR-503 was able to increase the cisplatin sensitivity of A549/DDP. After treatment with miR-503, the reverse folds (RF) to cisplatin was 2.48 fold, the intracellular level of Rh-123 was 2.49 fold, the apoptosis rate was 10.3 fold, the expressions of several drug-resistant related proteins, such as MDR1, MRP1, ERCC1, Survivin and Bcl-2 were downregulated significantly, as shown by WB, in contrast, the level of RhoE was elevated, the mRNA epression of MDR1 was 18.5%, the mRNA epression of MRP1 was 22.3%, the mRNA epression of ERCC1 was 18.6%, the mRNA epression of Survivin was 42.8%, the mRNA expression of Bcl-2 was 68.1%, the mRNA epression of RhoE was 206.5%, in addition, the phosphorylation of Akt decreased and transcriptional activities of NF-κB was 53.7%, AP-1 was 47.4% compared with control group.

**Conclusion:**

MiR-503 was able to reverse the cisplatin resistance of A549/DDP. MiR-503 processed this kind of effect by inhibiting the drug efflux, downregulating the expression of drug-resistant related proteins and promoting cell apoptosis.

肺癌是世界范围内最常见的恶性肿瘤之一，每年肺癌造成大约560, 000死亡，其中一半来自中国^[1]^。由于起病隐匿，早期少有症状或症状不明显，大多数肺癌患者在确诊时往往已到了晚期。近年来，由于治疗方案的不断优化，以及靶向药物的投入使用，肺癌的治疗现状已经得到了明显的改善，但仍然存在一些急需解决的问题，例如肿瘤细胞对药物的耐受性^[2]^。顺铂通过靶向DNA和拓扑异构酶Ⅱ（Topo Ⅱ），抑制DNA的合成和转录，诱导细胞凋亡，最终阻止肿瘤细胞生长^[3]^。在临床运用过程中，肺癌细胞往往发展出对顺铂的耐药性，使得药效明显下降。因此，探讨肿瘤细胞的耐药机制，开发新的逆转耐药性的方法，对提高临床患者的受益有十分重要的意义。已有的研究^[4-7]^发现，肿瘤细胞产生耐药的机制包括减少药物的吸收，通过ABC（ATP-binding cassette）转运蛋白增加药物的外排，通过谷胱甘肽系统增加对抗肿瘤药物的解毒作用，凋亡途径异常减少肿瘤细胞的凋亡^[4-7]^。

MiRNAs是一类内源性的不编码蛋白的小RNAs，长度一般为18个-24个核苷酸，可通过与目标mRNA的互补序列相结合，在后转录水平对基因的表达进行调控，导致目标mRNA的降解或者基因沉默^[8]^。很多研究^[9, 10]^已经提供了特异性miRNAs的表达与肿瘤密切相关的证据，这些研究清楚的展现出一个单独的miRNA可通过其调控的目标基因，对多种与肿瘤细胞失控生长、抗凋亡、迁移和侵袭，甚至是肿瘤细胞对药物治疗的应答产生调控作用。

最近，miRNA-503（miR-503）在癌症中的作用备受关注。研究发现miR-503在口腔癌和肝细胞癌中的表达下调^[11, 12]^，但在甲状旁腺癌和肾上腺皮质癌中却过表达^[13, 14]^。并且，在肾上腺癌中，miR-503上调与患者的总体生存期缩短有关^[13]^。在肝癌细胞系HCCLM3中，miR-503则可诱导细胞停滞于G1期并抑制细胞的迁移和侵袭^[11]^。然而，miR-503在肺癌耐药性中的作用尚无太多的研究。

在本研究中，我们发现miR-503的高表达可以逆转A549/DDP的耐药性，抑制该细胞的生长并促进其凋亡。我们的结果说明miR-503在肺癌顺铂耐药中发挥重要作用，其机制可能是通过调控多种与耐药相关蛋白，细胞增殖相关蛋白和凋亡相关蛋白等有关。

## 1 材料与方法

### 1.1 主要试剂与仪器

人肺癌细胞系A549和A549/DDP购自中国科学院上海生命科学研究院细胞资源中心；细胞培养基购自Gibco；可稳定表达miR-503的miR-503 precursor质粒（pre-miR-503）及其阴性对照质粒购自Invitrogen；TaqMan MicroRNA assay kit购自Applied Biosystems；Lipofectamine 2000购自Gibco；MTS、Rh-123、RT-PCR试剂盒，pGL4.74[hRluc/TK]质粒，pGL4.32(luc2P/NF-κB-RE/Hygro)质粒，pGL 4.44[luc2P/AP1 RE/Hygro]和Dual-GloTM Luciferase assay system均购自Promega公司；细胞凋亡流式检测试剂盒购自BD biosciences；单克隆抗体购自Santa Cruz公司；ECL免疫印迹底物试剂盒购自Millipore；顺铂购自Sigma公司；流式细胞仪：BD公司，酶标仪：Thermo，PCR仪：Thermo。

### 1.2 细胞培养与miR-503转染

A549和A549/DDP细胞培养于10 cm培养皿，37 ℃、5% CO_2_、饱和湿度的培养箱中，培养基为90% EMEM，10%胎牛血清。0.25%胰酶-EDTA消化传代，所有试验均采用对数生长期细胞。将A549/DDP细胞暴露于底剂量[1/10的半数抑制浓度（50% concentration of inhibition, IC_50_）]的顺铂中维持细胞耐药性。

A549/DDP培养于10 cm培养皿中，待其达到约80%融合时，按照试剂盒说明书的操作方法，加入100 nmol/L pre-miR-503质粒或者阴性对照质粒进行转染，继续培养48 h。传代培养后，取细胞裂解后，用TaqMan MicroRNA assay kit进行Q-PCR实验，定量检测miR-503，以评估pre-miR-503的转染效果。

### 1.3 MTS法检测miR-503对细胞耐药性的作用

取对数生长期的细胞，以2×10^4^/mL接种到96孔微孔板中，100 μL/孔，培养过夜使细胞贴壁。向对应试验孔加入不同浓度的顺铂，继续培养72 h，吸去培养基，加入100 μL含0.5 mg/mL MTS的RPMI-1640，继续培养4 h。最后用酶标仪测定490 nm波长下的光密度（optical density, OD）值，并计算药物对细胞的抑制率。抑制率＝（1-实验组OD值/对照组OD值）×100%。以顺铂浓度为横坐标，抑制率为纵坐标作图并拟合抑制曲线，求得IC_50_值。逆转倍数（reversal fold, RF）= IC_50_（无miR-503）/IC_50_（有miR-503）。

### 1.4 流式细胞术检测A549/DDP细胞内Rh-123含量及细胞凋亡

取对数生长期的经pre-miR-503质粒转染的A549/DDP细胞，加入10 μmol/L Rh-123染液，培养1 h后收集细胞，调整细胞浓度至10^6^/mL，用流式细胞仪检测细胞中Rh-123的荧光强度（488 nm激发光，560 nm发射光），以此检测细胞中Rh-123的含量。细胞凋亡采用PI/Annexin V-FITC双染法，均根据试剂盒说明书操作。以阴性对照质粒转染的肿瘤细胞作为对照。

### 1.5 Western blot法检测A549/DDP细胞中多种蛋白的表达

取对数生长期的经pre-miR-503转染的A549/DDP细胞，收集细胞裂解提取蛋白。BCA法测定细胞裂解物的蛋白含量，取等量蛋白质以12% SDS-PAGE法分离并转移至PVDF膜上，以单克隆抗体4 ℃过夜孵育以检测目标蛋白。洗去一抗，以HRP连接的二抗于室温孵育2 h，洗涤后以ECL试剂盒显示免疫印迹条带。α-tubulin作为内参。

### 1.6 Real-time PCR检测A549/DDP肿瘤细胞中多种蛋白基因的mRNA水平

取对数生长期的经pre-miR-503转染的A549/DDP细胞，用Trizol法提取各组总RNA，用Real-time PCR试剂盒进行逆转录得到cDNA。MDR1上游引物序列：5' -AAAAAGATCAACTCGTACCACTC-3' ，下游引物序列：5' -GCACAAAATACACCAACAA-3' ；MRP1上游引物序列5′-ACTTCCACATCTGCTTCGTCAGT-3，下游引物序列：5′-ATTCAGCCACAGGAGGTAGAGAGC-3′；ERCC1上游引物序列：5′-GGGAATTTGGCGACGTAATTC-3′，下游引物序列：5′-GCGGAGGCT-GAGGAACAG -3′；RhoE上游引物序列：5′-CCTCCACGTTGATTCGACTGTT-3′，下游引物序列：5′-TGTAAAAGCCG-TACGTTGCGGT-3′；Survivin上游引物序列：5′-GCATGGGTGCCCCGACGTTG-3′，下游引物序列：5′-GCTCCGGCCAGAGGCCTCAA -3′；Bcl-2上游引物序列：5′-ACGGGGTGAACTGGGGGAGGA-3′，下游引物序列：5′-TGTTTGGGGCAGGCATGTTGACTT-3′；β-actin上游引物序列：5′-TGAGCGCGGCTACAGCTT-3′，下游引物序列：5′-TCCTTAATGTCACGCACGATTT-3′；94 ℃变性3 min后，按下述条件扩增36个循环：95 ℃ 15 s，65 ℃ 30 s，72 ℃ 95 s，72 ℃延伸5 min。

### 1.7 数据统计

实验数据以Mean±SD表示，使用SPSS 13.0软件进行分析。采用单因素方差分析（*One-way ANOVA*）进行比较，以*P* < 0.05表示差异具有统计学意义。

## 2 结果

### 2.1 成功构建miR-503高表达的A549/DDP

如图 1所示，与对照组（A549/DDP细胞中转染空载质粒）和未处理的原发A549/DDP细胞相比较，pre-miR-503转染的A549/DDP细胞中，miR-503的表达水平明显上升，这一结果说明miR-503高表达的A549/DDP构建成功。

**1:**
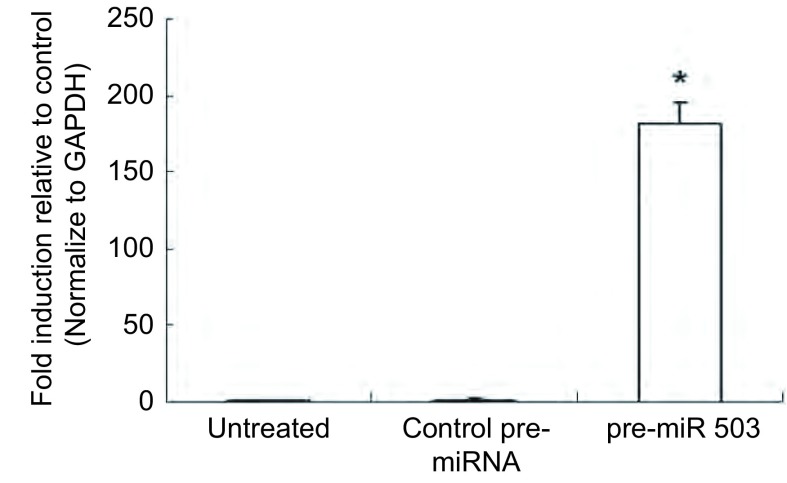
MiR-503在人肺癌细胞系中的表达。在A549/DDP细胞中转染pre-miR-503质粒48 h后，利用TaqMan MicroRNA分析试剂盒，通过Q-PCR检测miR-503的表达水平。未处理组：A549/DDP细胞；对照组：转染空白对照质粒的A549/DDP细胞；miR-503组：转染pre-miR-503质粒的A549/DDP细胞。实验数据以均数±标准差表示，*n*=10，^*^表示与对照组相比，*P* < 0.05。 The expression of miR-503 in human lung cancer cell lines. After transfection pre-miR-503 plasmids into A549/DDP cells for 48 h, the expression of miR-503 was detected by Q-PCR using TaqMan MicroRNA assay kit. Untreated group: the primary A549/DDP cells; Control group: A549/DDP cells transfected with control blank vector; miR-503 group: A549/DDP cell transfected with pre-miR-503 plasmids. Data was represented as Mean±SD, *n*=10, bars indicate SD, ^*^compared to the control group (*P* < 0.05).

### 2.2 转染miR-503可逆转A549/DDP的耐药性，提高胞内Rh-123浓度，促进细胞凋亡

实验结果显示，与对照组相比较，pre-miR-503转染的细胞对顺铂的敏感性明显增加，pre-miR-503组IC_50_为17.6 μM，其逆转系数是对照组（IC_50_为43.7 μM）的2.48倍；进一步研究发现miR-503转染组细胞吸收荧光染料Rh-123的能力较对照组提高了2.49倍，细胞凋亡率是对照组的10.3倍(图 2)。而且这些变化均与miR-503转染构成量效关系。

**2:**
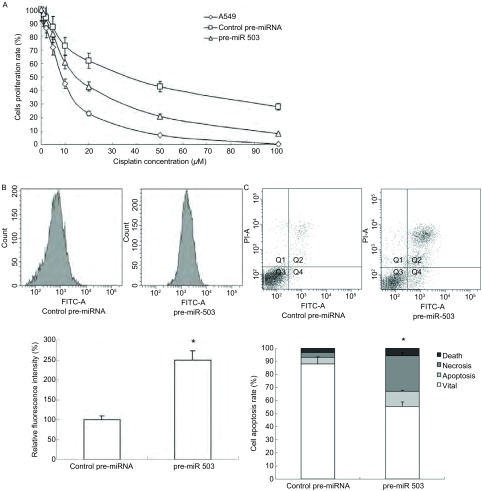
A549/DDP细胞中miR-503对药物敏感性，Rh-123的胞内浓度及细胞凋亡的作用。A：MTS法结果显示与对照组相比，转染pre-miR-503后，细胞对顺铂的敏感性明显增加，数据以Mean±SD表示，*n*=10，^*^表示与对照组相比，*P* < 0.05；B：流式细胞术结果表明与对照组相比，转染pre-miR-503后，细胞内Rh-123的浓度增加，数据以Mean±SD表示，*n*=10，^*^表示与对照组相比，*P* < 0.05；C：流式细胞术结果表明与对照组相比，转染pre-miR-503后，细胞凋亡数目增加，数据以Mean±SD表示，*n*=10，^*^表示与对照组相比，*P* < 0.05。 The effect of miR-503 on drug sensitivity, intracellular level of Rh-123 and apoptosis in A549/DDP cells. A: The MTS assay indicated that the DDP-sensitivity in pre-miR-503 group was significantly increased while compared to the control group. Data was represented as Mean±SD, *n*=10, bars indicate SD, ^*^compared to the control group (*P* < 0.05); B: The flow cytometry assay results showed that there was an increased intracellular level of Rh-123 in pre-miR-503 group compared with control group. Data was represented as Mean±SD, *n*=10, bars indicate SD, ^*^compared to the control group (*P* < 0.05); C: The flow cytometry assay results showed that there was an increased apoptosis in pre-miR-503 group compared with control group. Data was represented as Mean±SD, *n*=10, bars indicate SD, ^*^compared to the control group (*P* < 0.05).

### 2.3 MiR-503下调MDR1、MRP1、ERCC1、Survivin和Bcl-2的表达，上调RhoE的表达

为了进一步探讨miR-503逆转A549/DDP耐药性，促进肿瘤细胞凋亡的可能分子机制，我们分析了多种与耐药相关基因的表达变化情况。Western blot检测结果显示，与对照组相比较，pre-miR-503转染组细胞中MDR1和MRP1这两个多药耐药基因和与DNA损伤修复相关基因*ERCC1*的蛋白的表达水平均明显下调。此外，与肿瘤凋亡抑制相关的Survivin和Bcl-2的蛋白表达亦同时明显下调。有趣的是，miR-503上调了RhoE蛋白的表达水平（图 3）。进一步应用RT-PCR检测显示，在pre-miR-503转染组细胞中，*MDR1*、*MRP1*、*ERCCI*、*Survivin*和*Bcl-2*等基因的mRNA表达水平明显下调，仅分别是对照组的18.5%、22.3%、18.6%、42.8%和68.1%（*P* < 0.05），而RhoE mRNA表达则是对照组的206.5%，明显升高（*P* < 0.05）。这些结果表明miR-503对上述基因表达的调控是通过转录水平实现的。

**3:**
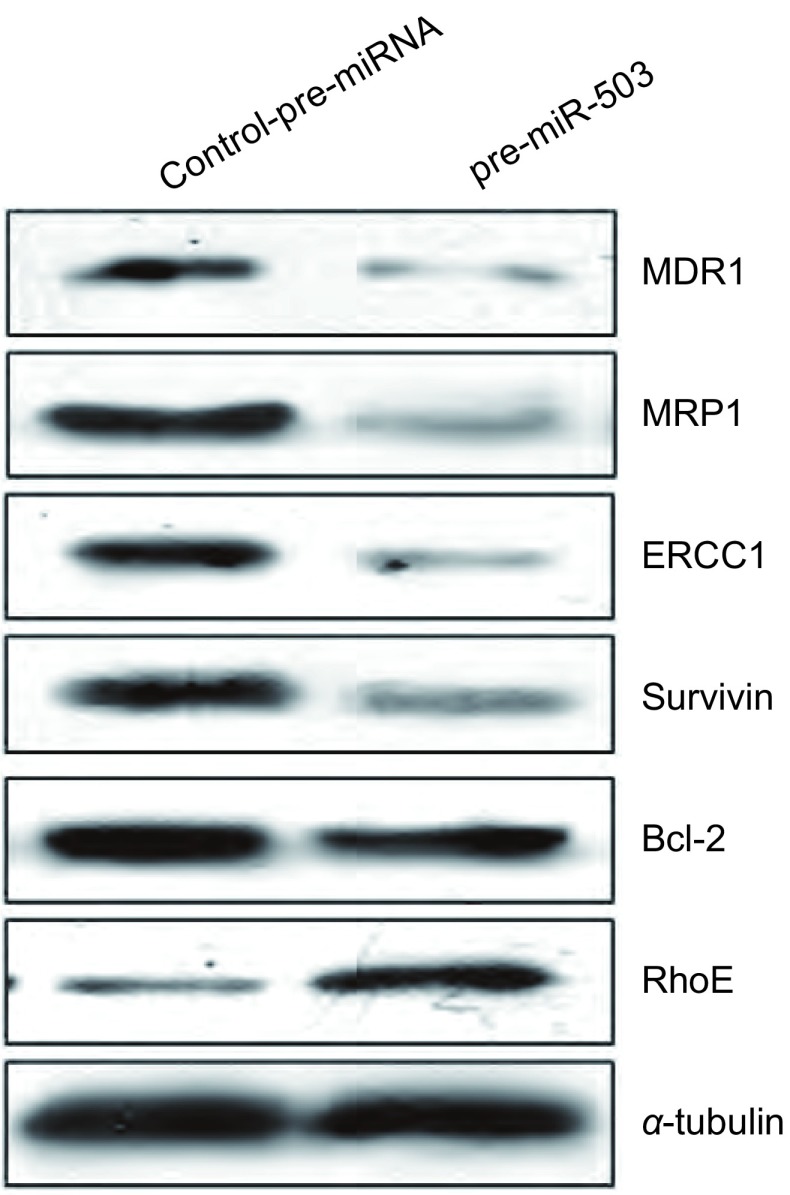
A549/DDP细胞中转染pre-miR-503后，耐药相关基因的蛋白表达水平。Western blot结果显示，与对照组相比，MDR1、MRP1、ERCC1、Survivin和Bcl-2的蛋白水平明显降低，而RhoE的蛋白水平明显增加，*α*-tubulin为内参。 The expression of different drug-resistance related genes in A549/DDP cells transfected with pre-miR-503. Compared to the control group, the expression of MDR1, MRP1, ERCC1, Survivin and Bcl-2 were dramatically down-regulated while the expression of RhoE was significantly up-regulated in pre-miR-503 group by western blot assay. *α*-tubulin was used as an internal control.

### 2.4 MiR-503抑制Akt的磷酸化

为了研究miR-503逆转A549/DDP耐药性的分子机制，我们重点研究了PI3K/Ak通路。Western blot显示，pre-miR-503转染后，Akt的磷酸化水平下降，说明PI3K/Akt信号通路受抑制（图 4）。

**4:**
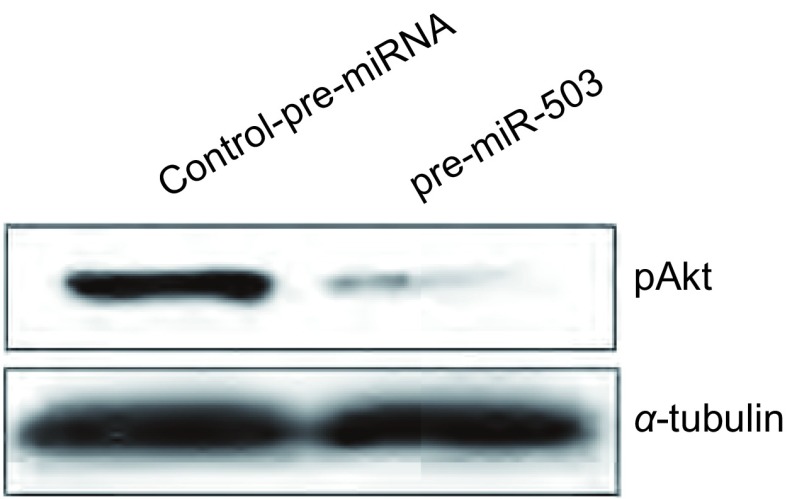
MiR-503对Akt磷酸化水平的影响。Western blot结果显示，与对照组相比，转染pre-miR-503质粒后，Akt磷酸化水平降低，*α*-tubulin为内参。 The effect of miR-503 on phosphorylation of Akt in human lung cancer cell lines. Western blot assay showed that the phosphorylation of Akt was downregulated in pre-miR-503 group compared with control group. *α*-tubulin was used as an internal control.

## 3 讨论

肿瘤细胞增加药物的外排，减少药物的吸收是其耐受化疗药物的一种主要手段。ABC家族的跨膜转运蛋白，例如MDR1和MRP1，可将药物从细胞内测泵到外侧，在耐药的肿瘤细胞中经常过表达^[15]^。Pre-miR-503转染后，流式细胞术检测可见细胞内Rhodamin-123的含量升高，间接说明miR-503逆转A549/DDP的耐药性与减少药物的外排有关。进一步的研究发现，miR-503下调了MDR1和MRP1蛋白水平与mRNA水平的表达，为上述假设提供了佐证。

除了MDR1和MRP1，其他分子也可能参与肿瘤细胞耐受顺铂有关。ERCC1可以修复化疗药物造成的DNA损坏^[16-18]^，因此它的表达水平很可能与A549/DDP的耐药性有关。Western blot显示，A549/DDP细胞中ERCC1具有较高的表达，而pre-miR-503处理后其表达水平明显下降，并且这种下降与mRNA的下调有关。RhoE是一个非典型的RhoGTPase家族成员，它一直处于活化的GTP结合状态，最近发现其在一些主要的肺癌细胞系和癌组织中的表达下调，可促进肿瘤细胞的侵袭和转移^[19]^。在本研究中，miR-503可上调RhoE的表达水平，可能对肿瘤起到抑制的作用。

Survivin是凋亡抑制蛋白家族的成员，具有肿瘤特异性，只表达于肿瘤和胚胎组织中，它可抑制肿瘤细胞的凋亡，促进增殖和血管新生，因此被认为是一个具有很高价值的肿瘤治疗靶点^[20, 21]^。实验结果显示，miR-503可下调Survivin表达，这与miR-503增加肿瘤细胞的凋亡率相匹配。Bcl-2是一个在细胞凋亡中发挥重要作用的蛋白，可抑制肿瘤细胞的凋亡，在很多肿瘤中发现Bcl-2的高表达与肿瘤的耐药密切相关^[22, 23]^。已有研究^[24]^显示，在非小细胞耐顺铂细胞系A549/CDDP中，miR-503可以下调Bcl-2的表达，逆转肿瘤的耐药性。本研究发现，在A549/DDP细胞中，Bcl-2的蛋白表达水平明显升高，说明Bcl-2介导的抗凋亡是肿瘤产生耐药的主要机制之一。而pre-miR-503转染可使A549/DDP细胞Bcl-2的表达明显下降，这与流式观察到的细胞凋亡率上升一致，是miR-503逆转肿瘤耐药性的重要机制之一。

本研究发现，miR-503对细胞耐药性逆转起着重要调节作用的信号通路是PI3K/Akt^[25-27]^，miR-503可抑制Akt的磷酸化，抑制PI3K/Akt信号通路的活性，从而抑制肿瘤细胞的增殖，逆转其抗药性，虽然具体的机制有待进一步的研究，但这种负调控可能是miR-503逆转A549/DDP耐药性的手段之一。
